# Venereal Prevention

**Published:** 1920-07-10

**Authors:** 


### VENEREAL PREVENTION.

SOME eighteen months after publication of the original straightforward letter signed by the late Sir William Osler and some of the most distinguished members of the medical profession, Dr. Addison last week apparently arrived at yet another official indecision on the question of venereal disease. A deputation representing the Society for the Prevention of Venereal Disease submitted to him the following resolution as unanimously passed at its recent annual meeting.

"That inasmuch as the Ministry of Health has failed, and public bodies, including the L.C.C., have declined to provide the means of delayed disinfection against venereal disease at ablution centres, this meeting calls upon the Ministry of Health and upon local authorities to instruct all qualified chemists to sell such means of immediate self-disinfection against venereal disease as may be approved from time to time by the Ministry of Health or by the medical officers of health."

In reply to this suggestion, which in practice would, in part at least, amount to an official recognition of a trade which is already widely carried on, the Minister of Health expressed a series of views which give the broad impression that in any circumstances those responsible for the present active campaign on venereal matters will have for some time to wait and watch developments before they can expect any new forms of Governmental assistance to take shape. When the venereal question first came prominently before society, in early 1919, we took the opportunity to point out that the differences of opinion between the two committees voluntarily interesting themselves in the matter were not concerned with the effectiveness of the medical measures themselves. The question was simply that venereal disease could be prevented by the use of certain agents *before* infection, and less certainly by its use within an hour or two *after* infection. The Society advocated the former, the National Council supported the latter. Now through a great maze of arguments we have come down to the situation sufficiently described in the resolution quoted above. Official recognition and assistance has concentrated itself meanwhile upon the organisation of venereal clinics, which are doing invaluable work, but it has let the campaign of social instruction and enlightenment proceed largely through the agency of others.

And still, at the end of eighteen months, the latest attempt to get a definitely sanctioned reform is met by the same "expectant" attitude. But in the reply itself there are statements which do not encourage the belief that waiting is necessarily the best policy. It is not helpful to read that we do not know whether venereal disease is increasing or not. Nor is a very progressive spirit foreshadowed in the statement that we have even as yet insufficient basis of ascertained fact "upon which it is quite safe to dogmatise." The deputation were told that they must first have "an overwhelming case" before action can be justified and conflict with opinion avoided. But in reply it might be said that for this very reason the various situations previously under discussion have been perforce progressively weaker modifications of Sir William Osler's original policy of direct action, and if the same process continues indefinitely, it is difficult to see how any of the obviously well-meant and excellent advice which he gave can ever be put into practice.

The probability of a "great wave of public feeling" which is so greatly feared may perhaps become less as the social campaign of education proceeds, and perhaps Dr. Addison may think that in a few years time the public will be ready to receive these reforms without indignation against any of their originators—or even against the Minister of Health. Nevertheless, we have meanwhile our crowded clinics, and therefore, demonstrated by their very congestion, an appalling incidence of the disease.

